# Current perspectives on neuromodulation in ALS patients: A systematic review and meta-analysis

**DOI:** 10.1371/journal.pone.0300671

**Published:** 2024-03-29

**Authors:** Ana M. Jiménez-García, Gaspard Bonnel, Alicia Álvarez-Mota, Natalia Arias

**Affiliations:** 1 BRABE Group, Department of Psychology, Faculty of Life and Natural Sciences, University of Nebrija, Madrid, Spain; 2 Health Research Institute of the Principality of Asturias (Instituto de Investigación Universitaria del Principado de Asturias), Oviedo, Spain; 3 INEUROPA, Instituto de Neurociencias del Principado de Asturias, Plaza Feijoo, Oviedo, Spain; Anadolu University: Anadolu Universitesi, TURKEY

## Abstract

Amyotrophic Lateral Sclerosis (ALS) is a progressive neurodegenerative disease that affects motor neurons, resulting in muscle weakness, paralysis, and eventually patient mortality. In recent years, neuromodulation techniques have emerged as promising potential therapeutic approaches to slow disease progression and improve the quality of life of ALS patients. A systematic review was conducted until August 8, 2023, to evaluate the neuromodulation methods used and their potential in the treatment of ALS. The search strategy was applied in the Cochrane Central database, incorporating results from other databases such as PubMed, Embase, CTgov, CINAHL, and ICTRP. Following the exclusion of papers that did not fulfil the inclusion criteria, a total of 2090 records were found, leaving a total of 10 studies. R software was used to conduct meta-analyses based on the effect sizes between the experimental and control groups. This revealed differences in muscle stretch measures with manual muscle testing (p = 0.012) and resting motor threshold (p = 0.0457), but not with voluntary isometric contraction (p = 0.1883). The functionality of ALS was also different (p = 0.007), but not the quality of life. Although intracortical facilitation was not seen in motor cortex 1 (M1) (p = 0.1338), short-interval intracortical inhibition of M1 was significant (p = 0.0001). BDNF showed no differences that were statistically significant (p = 0.2297). Neuromodulation-based treatments are proposed as a promising therapeutic approach for ALS that can produce effects on muscle function, spasticity, and intracortical connections through electrical, magnetic, and photonic stimulation. Photobiomodulation stands out as an innovative approach that uses specific wavelengths to influence mitochondria, with the aim of improving mitochondrial function and reducing excitotoxicity. The lack of reliable placebo controls and the variation in stimulation frequency are some of the drawbacks of neuromodulation.

## Introduction

Amyotrophic lateral sclerosis (ALS) continues to be a fatal neurodegenerative disease. ALS has a life expectancy of two to five years, although exceptional cases reaching up to ten years have been found due to the variability of the disease. The disease is accompanied by symptoms which are often collectively termed ‘non-motor’ or ‘extra-motor’ [1]. Common but varying symptoms include, generally, a deterioration of the muscles that control our extremities (hands, arms, and feet) and a degeneration in the muscles and fibres in charge of speech, swallowing, and even breathing, which represents a challenging living situation for the patients and their careers [2]. Non-motor symptoms include cognitive dysfunction which is a common non-motor manifestation in ALS, affecting 30–50% of individuals, and approximately 15% meet criteria for frontotemporal dementia (FTD) [3–5]. ALS-related cognitive impairment manifests through executive dysfunction, language deficits, and challenges in social cognition, with a notable prevalence of apathy as a prominent behavioral change [6–9]. Moreover, Results from a recent population-based study in Scotland indicated a 19.7% prevalence of neuropsychiatric disorders in individuals with ALS. Mood disorders accounted for 70%, and neurotic disorders, including anxiety, stress-related, and somatoform disorders, constituted 31.67% of these cases [10]. Furthermore, results from a recent population-based study in Scotland indicated a 19.7% prevalence of neuropsychiatric disorders in individuals with ALS. Mood disorders accounted for 70%, and neurotic disorders, including anxiety, stress-related, and somatoform disorders, constituted 31.67% of these cases [10].

No available full-recovery treatment has been found yet, and only drug use of riluzole is known to treat and alleviate part of the ALS symptomatology. Riluzole, as a glutamate antagonist, inhibits neural excitability by blocking the excessive release of glutamate from motor neurons, meaning only motor outcomes such as dysphagia (seen in bulbar-onset and limb-onset) or cramping of the limbs can be treated, with a very minimum amount of evidence on improvements in fatigue, emotional stability (untimely crying or laughing), or behavioural changes. It is important to note that riluzole only slows down the progression of ALS. Also, the effectiveness of the drug has been proven to be at its maximum efficacy at the first and last stages of the disease, with little to no effect found on phases 2 and 3 [11, 12], which begs the question of alternative treatments for the relief and commodities of the patients that aren’t covered by drug use. Lately, a new drug, Edaravone, has been shown to be effective in mitigating oxidative injury by acting as a reactive oxygen species scavenger and inhibiting peroxyl radical (LOO*)-induced peroxidation systems [13] in central nervous system neurons, principally motor neurons at risk for degeneration in ALS [14]. Also, a Japanese phase III confirmatory study has shown a slow decline in ALS functioning rating scale-revised (ALSFRS-R) scores in the edaravone-treated patients as compared with placebo [15–17]; however, the mechanism is still unknown.

In this line, gene therapy has been proposed as a potential target of the disease; however, due to the new transcriptions of RNA generated in ALS within the system and the number of cells already damaged, the effectiveness of this therapy is less than initially expected [18]. Under this scenario, patients are left with devices for assisted respiration that only offer commodities rather than solutions as the disease progresses into its final stages, such as a mechanical ventilator for assisted respiration when the patient cannot do it on its own or being connected 24 hours a day to a machine via a tracheotomy.

Alternatives for treatment have been a work in progress during these years, opening up the idea of neuromodulation as a potential non-invasive treatment which refers to methods or procedures that do not involve penetration or disruption of the tissues, thereby avoiding direct physical contact with internal structures. Non-invasive techniques are typically applied externally to the body, minimizing the need for surgical intervention and reducing the associated risks and recovery time. In that sense, neuromodulation aims to stop the progression of ALS while treating the common symptomatology of the disease and offers a non-invasive alternative among its various options that aims to find a less aggressive and more accessible treatment option and care for patients. Neuromodulation has been introduced as the process via which alteration of the central, peripheral, or autonomic nervous systems can be inhibited, stimulated, modified, regulated, or even offered as a therapeutic alteration of activity [19]. As aforementioned, neuromodulation offers non-invasive procedures, such as magnetic, electrical, or optical stimulation, but also considers minimally invasive procedures like deep brain stimulation (DBS) [20].

Regarding the neuromodulatory tools applied in ALS, transcranial magnetic stimulation (TMS), and especially repetitive TMS (rTMS), has gained traction as it relies on the principle of electromagnetic induction, with a time-varying magnetic field leading to electrical current changes in the brain [21] which is able to produce inhibitory or excitatory effects on neural excitability by emitting repeated trains of pulses at low to high frequencies (1–10 Hz), making this especially relevant for ALS treatment. The inhibition of neural excitability signifies a decrease in glutamatergic excitoxicity [22], which is considered one of the main causes of degeneration in the system. TMS serves as a modality for eliciting cerebral electrical activity through the application of magnetic pulses on the scalp. This approach offers an advantage over direct electrical stimulation on the scalp, as the magnetic field generated by TMS can seamlessly traverse the scalp and skull to reach the brain, in contrast to electricity [23]. In the TMS paradigm, alterations in the electric field produced by the stimulating coil’s current generate a time-varying magnetic field, initiating electromagnetic induction. This process results in the generation of an electrical current, specifically within the cortical region of the brain, in the context of TMS for brain stimulation [24].

Several investigations have documented an initial decrease in motor threshold (MT), succeeded by a gradual development of cortical inexcitability in later stages [25–28]. A recent prospective study involving a substantial ALS patient cohort (n = 345), characterized by mild to moderate disease severity [mean ALS Functional Rating Scale (ALSFRS-R): 40.5] during neurophysiological evaluation, reveals an augmentation of cortical hyperexcitability with prolonged disease duration. However, subjects exhibiting inexcitable primary motor cortex (M1) to transcranial magnetic stimulation (TMS) were excluded in this study [29]. It is noteworthy that a substantial reduction in excitability or complete inexcitability to TMS was identified in 21% of ALS patients in this investigation. Moreover, TMS has been proven to control the phase in which the patient may be, which helps with treatment and prognosis as it can identify a reduction of motor-evoked potentials (MEP), motor threshold, and central motor conduction time. It is mentioned in this same article that the reduction of MEP caught by TMS can be considered a biomarker of ALS progression. TMS is also mentioned to recognise abnormal or lesser-known phenotypes, such as Progressive Muscular Atrophy [30].

Furthermore, accelerated high-frequency rTMS called theta burst stimulation (TBS) is also considered on the rise as it creates similar suppressing and facilitating neuromodulation effects to TMS but consists of shorter sessions and lower modulatory intensities [31]. The impact of continuous, cTBS, in ALS was investigated in four studies conducted by Di Lazzaro et al. In an initial randomized trial involving 20 ALS patients, active bilateral cTBS applied to the primary motor cortex (M1) for 5 days per month exhibited an association with a deceleration of disease progression after 6 months of treatment [22]. However, this outcome was not replicated in a subsequent trial that extended the duration of cTBS treatment to 12 months [32]. In a further small study by the same group [33], where the cTBS dosage was doubled to 10 days per month, a trend toward a slower progression over 6 months was observed when compared with pooled data from patients treated for 5 days per month and "sham"-treated patients in previous studies [22, 32]. Although it was not possible to demonstrate a reduction from baseline progression in this new patient group [33]. Two additional studies with cTBS were small case series, one involving stimulation over a prolonged period of over 2 years in one subject [34] and the other comprising a 1-year open-label extension study in three subjects previously observed for 1 year under placebo stimulation [35]. In both cases, a reduction in ALS Functional Rating Scale-Revised (ALSFRS-R) score was observed in comparison with baseline observations. The most common side effect of rTMS has been reported to be mild headaches, with no harmful cognitive effects known to date.

Transcranial direct current stimulation (tDCS) is one of the most widely used methods of Transcranial electrical stimulation (tES) which aims to alter brain function in a non-invasive way, by applying different types of current to electrodes on the scalp [36–38]. tDCS employs multiple electrodes, including an anode and cathode, delivering mild currents (ranging between 1 and 2 mA) across the scalp. These low-intensity currents lead to depolarization or hyperpolarization of neuron membranes, thereby altering their response thresholds and synaptic efficiency [37, 38]. tDCS has been broadly applied and consists of the placing of an anode, which leads to stimulation, and a cathode, which induces inhibition, over the scalp of the patient, whose function is determined by the polarity of the stimulation. Thus, tDCS manages intracortical excitability with long-lasting effects when applied for a very short amount of time [39]. However, when applied to ALS patients, little to no effect has been demonstrated in some trials, as in Quarterone et al. [39] and Munneke et al. [40]. The proposed reasons range from the altered glutamate transmission to the fact that the frequency of tDCS is too low for ALS patients, who are said to have a higher threshold than able-bodied patients. Studies performed later by Sánchez-Kuhn et al. [41] have proved that stimulation at a higher intensity (2 mA, 20 min during 5 consecutive days) showed significant improvement in sensory deficits like spatial discrimination as well as pain relief in the patient. Benussi et al. [42] explored a more intricate transcranial direct current stimulation (tDCS) paradigm, involving concurrent bi-anodal motor cortex and cathodal spinal stimulation (corticospinal tDCS), seeking a synergistic effect. The randomized controlled trial included 30 patients who received real tDCS for 2 weeks, showing a significant improvement or stabilization in muscle strength, quality of life scores, and caregiver burden. This positive impact persisted at the 6-month follow-up, accompanied by the restoration of transcranial magnetic stimulation (TMS) parameters related to intracortical circuit excitability (short-interval intracortical inhibition and intracortical facilitation). Notably, both real and sham tDCS groups exhibited a lower-than-usual disease progression, reflected in a decline of ALS Functional Rating Scale-Revised (ALSFRS-R) score of <2 points over 6 months. The same group is currently conducting a further clinical trial (ClinicalTrials.gov ID: NCT04293484) to evaluate the stability of improvement after repeated treatment. One of the main benefits mentioned by Sánchez-Kuhn et al. [41] is its portability as it is a lightweight device, unlike TMS or rTMS, which require continuous assistance, and its combination with other therapies while undergoing treatment like motor rehabilitation, as well as its after-effects on the patient, which so far have been reported to be a light transient itchiness on the area of the scalp affected.

Another neuromodulatory technique is photobiomodulation (PBM), which is an approach that harnesses the therapeutic properties of various wavelengths of visible light based on the application of near-infrared (NIR) light. Red and NIR light have deep penetration abilities and therefore they pass through the skull and into the cortical surfaces of the brain. PBM non-invasive (trans-cranially) delivers photons from an external light source to the head and thence into the brain tissue and it has been demonstrated to not only have regenerative properties on pain relief, regenerative medicine, healing, prevention of tissue death, and reduction of inflamed areas but to provide beneficial effects on Alzheimer’s Disease (AD), Parkinson’s Disease (PD), and familial Amyotrophic Lateral Sclerosis (fALS) [43]. Longo et al. [44] have also found evidence that PBM could improve, even if temporarily, motor function, but more specifically, respiratory autonomy, to a certain degree. Much is yet to be studied on PBM, but research and trials conducted so far have shown promising results.

Another relevant technique within ALS is neuromuscular magnetic stimulation (NMMS), which can penetrate skin, fat, and bone to create a narrow electrical field, stimulating tissues deep within the neuromuscular system. The power of NMMS is that involves electrical stimulation without stimulating the skin nociceptors [45]. The technique consists of intramuscular stimulation of the axon tree via short but strong magnetic pulsations [46] so as to manage strong muscle contractions, even if muscle denervation occurs, which makes this relevant for ALS. Musarò et al. [45] applied NMMS to spinal on-set patients that showed counteraction of degeneration on fast twitch muscle fibres and modification of the acetylcholine receptor (AChr) response, which in turn improved ACh function, which is reduced in ALS. Also, preservation of muscle mass and, as such, counteracting muscle atrophy and attenuating muscle denervation in ALS patients have been observed [45].

Also, cervical transcutaneous spinal stimulation (cTSS) is a non-invasive technique that involves the application of electrical stimulation to the cervical region of the spinal cord through the skin. This technique utilizes transcutaneous electrical stimulation, targeting the cervical spinal nerves and associated neural pathways to modulate neuronal activity. This technique applied to ALS patients has been shown to activate motor functionality as it triggers afferent sensory circuits that are not affected by the disease [47]. Safety reports on cTSS included light-headedness, nausea, feeling flushed, neck pain, and a sensation of "sharp breathing", all of which disappeared within a minute or less after treatment.

Finally, therapeutic electrical stimulation (TES) refers to the application of controlled electrical currents to modulate neural activity in specific regions of the body. TES can be applied through electrodes placed on or near the skin, and the electrical currents delivered are tailored to achieve therapeutic effects, such as pain relief, muscle strengthening, or neural modulation. So,TES offers, via electrical stimulation, improvement of atrophy in the muscles targeted, a general decrease in spasticity, and an increase in muscle strength [48], all of which are especially beneficial in slowing the progression of ALS. Some of the limitations found when applying TES include the ineffectiveness of this treatment for denervated muscles. However, this technique showed improvement in separated applications of two cycles, such as a sudden improvement in the first month and another one later on [48], which can only be achieved by a rigorous dedication to the therapy, which some of the patients with ALS may not be able to do due to transportation or monetary issues.

It is also important to mention a very prominent limitation found in all the different techniques that neuromodulation offers: predictability and replicability of the response given all the factors that each device manages, such as age, gender, or genetic and psychological factors of the patient, as well as technical issues such as waveform parameters or timing of techniques [49]. These factors and the associated changes indicate that neuromodulatory techniques have not been thoroughly researched enough to provide a proper amount of evidence that indicates a full and detailed understanding of each technique and each individual factor. This cannot be blamed alone on the techniques themselves but also on the current knowledge of ALS. Another important issue found when applying these techniques to patients is that no standardised instrument has yet been able to measure the relationship that unites the medical condition, its own objective data, the subjective perspective of the patient, and consideration of benefits or harms.

In this scenario, a systematic review and meta-analysis have been considered relevant as it becomes more and more clear that research and trials are emerging as recently as this year (2023). The aim of this study is to evaluate the effects of different neuromodulation methods in ALS patients, between neuromodulation and control groups, in order to determine the efficacy and therapeutic potential of these interventions in improving the condition of ALS patients. This offers new and innovative perspectives on the different non-invasive alternative treatments for ALS. Also, this work may give a clearer view of active and already effective treatments that can be used for future research or as a basis for funding, which may have been overlooked.

## Methods

In this article, a systematic review of the scientific bibliography available until August 8, 2023, was done by two independent researchers to evaluate the neuromodulation methods used and usable for the treatment of amyotrophic lateral sclerosis (ALS). The Preferred Reporting Items for Systematic Reviews and Meta-Analyses (PRISMA) method for the research strategy was followed (S1 Text).

### Research strategy

For this research, the data base Cochrane Central was used, which included results from other data bases such as PubMed, Embase, CTgov (ClinicalTrials.gov), CINAHL (Cumulative Index to Nursing and Allied Health Literature) and ICTRP (International Clinical Trials Registry Platform), without any time limit as far as date of publication. The keywords selected for this research were the following: « Neuromodulation »,« DBS », « deep brain stimulation »,« VNS », « vagus nerve stimulation »,« FES », « functional electrical stimulation »,« EGS », « electrical gastric stimulation »,« Electric stimulation »,« rTMS », « recurrent transcranial magnetic stimulation »,« TES », « transcranial electric stimulation »,« tDCS », « transcranial direct current stimulation », « tACS », « transcranial alternating current stimulation », « tRNS », « transcranial random noise stimulation », « ONS », « optical nerve stimulation », « tPBM », « transcranial photobiomodulation »,« iTBS », « intermittent theta burst stimulation »,« ALS », « amyotrophic lateral sclerosis », « fALS », « familial amyotrophic lateral sclerosis », « sALS », « sporadic amyotrophic lateral sclerosis », « TDP-43 », « SOD1 », « FUS », « fused in sarcoma » and « C9orf72 sequence ». They were then paired up to formulate 2090 individual´s research.

No chronological, language, or methodological filters have been imposed on the search engines, and all resulting data sets were exported and compiled in an Excel document. The search strategy was further broadened to include screening references cited in relevant review articles.

### Study selection

Following the removal of duplicates, all remaining articles had their titles and abstracts screened for eligibility. Epidemiological studies and articles that did not specifically pertain to ALS or neuromodulation were deemed ineligible. After the initial screening phase, the full texts of selected studies were retrieved and reviewed in detail against the inclusion criteria. In order for a study to be included in the systematic review, it had to (i) show clear evidence of the application of neurodomulatory techniques such as electrical, magnetic, or optical, (ii) employ ALS or FTD patients, whether sporadic (sALS) or familial (fALS), and (iii) examine any cognitive or motor improvements or present findings that can be extrapolated to improvements in ALS pathology.

### Method quality

The methodological quality of the clinical research studies was assessed using the Cochrane Bias Risk Assessment Scale [50]. This systematic tool was used to assess the risk of bias in seven key domains: participant selection, randomization, allocation concealment, blinding of participants and staff, blinding of results, incomplete data handling, and report selection. Each domain was classified as having a low, uncertain, or high risk of bias, providing a comprehensive assessment of the methodological quality of individual studies. The application of this scale allowed for a rigorous assessment of the internal validity and reliability of the results obtained from the studies included in the review.

The Case Study Evaluation Tool (CaSE) was used to assess the methodological quality of the single-case studies [51]. The scale covers three main categories: methodology (7 items), clinical components (6 items), and theory (5 items). In methodology, CaSE assesses how the study was conducted, including the choice of method, description of participants, and research procedures, as well as ethical considerations. Clinical Components focuses on the clinical details of the study, such as patient history and condition, diagnosis, treatment, and therapy outcomes. Finally, in Theory, it examines the theory underpinning the study, including theoretical references, its application to clinical decision-making, and its relationship to the results. The CaSE is a useful tool to help reviewers assess the methodological quality and presentation of case studies, identifying areas of strength and weakness in the presentation of information.

### Meta-analysis

A continuous random effects model with a standard mean difference was employed to conduct the meta-analysis. Publications that reported (i) muscular changes such as muscle strength, maximum voluntary isometric contraction and resting motor threshold (ii) functionality in ALS, (iii) quality of life, (iv) changes in short latency intracortical inhibition or in (v) intracortical facilitation and (vi) used brain derived neurotrophic factor as biomarker underwent methodological quality assessment performed by two independent researchers to minimise the risk of bias. Studies were excluded from meta-analysis for being a single case such as Longo et al. [44] and Handa et al. [48] or for not being a longitudinal study such as Wu et al. [47]. Significance played no role in the selection process, with studies reporting null findings included by the experimenters. Authors of the relevant publications were not contacted directly regarding the raw data sets. Instead, numerical data was extracted directly from the figures using the online data extractor tool PlotDigitizer. Information regarding the figures used to calculate the different outcomes of meta-analysis is summarised in Table 1. Means, standard deviations and sample sizes were entered into R Studio software version 4.3.1. [52] which automatically calculated standard mean difference (SMD), confidence intervals (CIs), heterogeneity and overall effect size using a random effects model.

**Table 1:** Overview of the studies included for meta-analysis.

Study	Included	Description	Comment
Benussi et al. [42]	Yes	The effect size was calculated from the means and standard deviations of the variables ALSFRS-R, SICI, ICF, RMT, and QoL, which were taken directly from Table 2 included in the supplementary material.	Mean differences between the experimental group and the control group, as well as changes from the baseline up to 6 months, were considered for the presentation of the results.
Di Lazzaro et al. [2]	Yes	The effect size was calculated from the means and standard deviations of the variables ALSFRS-R, BDNF, and MMT, which were taken directly from the text of the results section.	Mean differences between the experimental group and the control group, as well as changes from the baseline up to five stimulation sessions, were considered for the presentation of the results.
Di Lazzaro et al. [32]	Yes	The effect size was calculated from the means and standard deviations, which were taken from Fig 1A (ALSFRS-R), 1B (MMT), and 1C (MVIC). In addition, and to confirm the data extracted from the figures, effect sizes were extracted using an R function, using as references the values of the F-statistic from ANOVA analysis and the sample size of the two groups. The effect size was calculated from the mean and standard deviation of BDNF, which were extracted from the text of the results section.	Mean differences between the experimental group and the placebo group, as well as changes during cycles regarding the baseline, were considered for the presentation of the results.
Di Lazzaro et al. [54]	Yes	Participants’ direct scores of MMT were extracted from Fig 1, which allows for the calculation of the mean and standard deviation of each group. The effect size was calculated from the means and standard deviations.	Mean differences between the experimental group and the placebo group, as well as changes during treatment regarding the baseline, were considered for the presentation of the results.
Handa et al. [48]	No	The authors do not report the means and standard deviations obtained in the MMT.	Changes in the participant’s direct score throughout the treatment were considered for the presentation of the results.
Longo et al. [44]	No	The authors do not report direct values of MMT.	Changes in the participant’s motricity measured through the travelled distance or functional skills (dressing, subjecting objects, among others) were considered in the presentation of the results.
Munneke et al. [40]	Yes	The effect size was calculated from the means and standard deviations, which were taken from Figs 1 (SICI) and 2 (ICF) in the supplementary material and from Fig 3 (RMT) in the manuscript. In addition, and to confirm the data extracted from the figures, effect sizes were extracted using an R function, using as references the values of the F-statistic from the ANOVA analysis and the sample size of the two groups.	Mean differences between the patients and the control group, as well as changes from the baseline up to 5 consecutive days of cTBS treatment, were considered for the presentation of the results.
Musarò et al. [45]	Yes	The means and standard deviations of MMT were taken directly from the text of the results section. The effect size was calculated from the means and standard deviations.	Mean differences between the experimental and control groups, as well as changes from T0 (first recording of clinical strength and neurophysiological parameters before stimulation), T1 (after one week of stimulation), and T2 (after two weeks of stimulation), were considered for the presentation of the results.
Wu et al. [47]	No	The authors report baseline levels of ALSFRS-R, but not those obtained after applying the protocol.	Tolerability responses, thresholds, and response latencies measured by participants after the 170 cTSS sessions were considered for the presentation of the results.
Zannette et al. [53]	Yes	Effect sizes of ALSFRS-R, MMT, QoL, and MVIC were extracted from Table 2 using an R function, using as references the values of the F-statistic from the ANOVA analysis and the sample size of the two groups.	Mean differences between the Active rTMS’ group and the Sham rTMS’ group, as well as changes from the baseline up to 20 trains of 15 stimuli sessions, were considered for the presentation of the results.

Note. ALSFRS = ALS functional rating scale; BDNF = Brain-derived neurotrophic factor; ICF = Intracortical facilitation; MMT = Manual Muscle Testing; MVIC = Maximum voluntary isometric contraction; N/A = Not Applicable; QoL = Quality of life; RMT = Resting motor threshold; SICI = Short-latency intracortical inhibition.

To obtain the effect size, the custom function ’calculate_d’ was used, which takes as input the means, standard deviations and sample sizes of two groups. When these data were not submitted by the authors, we used the function ’cat’ which takes the values of the F-statistic and the degrees of freedom provided by the authors to extract the effect size (S1 Code). The meta-analysis analysis was carried out using the ’rma.uni’ function of the ’metafor’ package, which calculates a random effects model to estimate the overall effect size and its confidence interval. The results of the analysis were printed using the ’print’ function, and a forest plot with the study names was generated using the ’forest’ function (S2 Code). Studies were weighted in the final analysis based on the precision of their data as determined by confidence intervals, with greater weights usually indicative of larger sample sizes. Finally, to analyse the robustness of the results, the code to calculate the Fail-Safe N based on the Rosenthal approach with the ’metafor’ package was used (S3 Code).

## Results

### Study selection

The database identified 2090 potential reports that could be included in this systematic analysis. After removing the duplicates, 854 reports were left for screening. After a look at the title, abstract and keyword list, 840 were excluded because they did not meet the inclusion criteria by not mentioning amyotrophic lateral sclerosis, a method of neuromodulation, by not being clinical trials or all three. One report was excluded because it wasn’t available in either english, spanish or french and five others because although meeting the inclusion criteria, they weren’t presenting any results.

**Fig 1:**
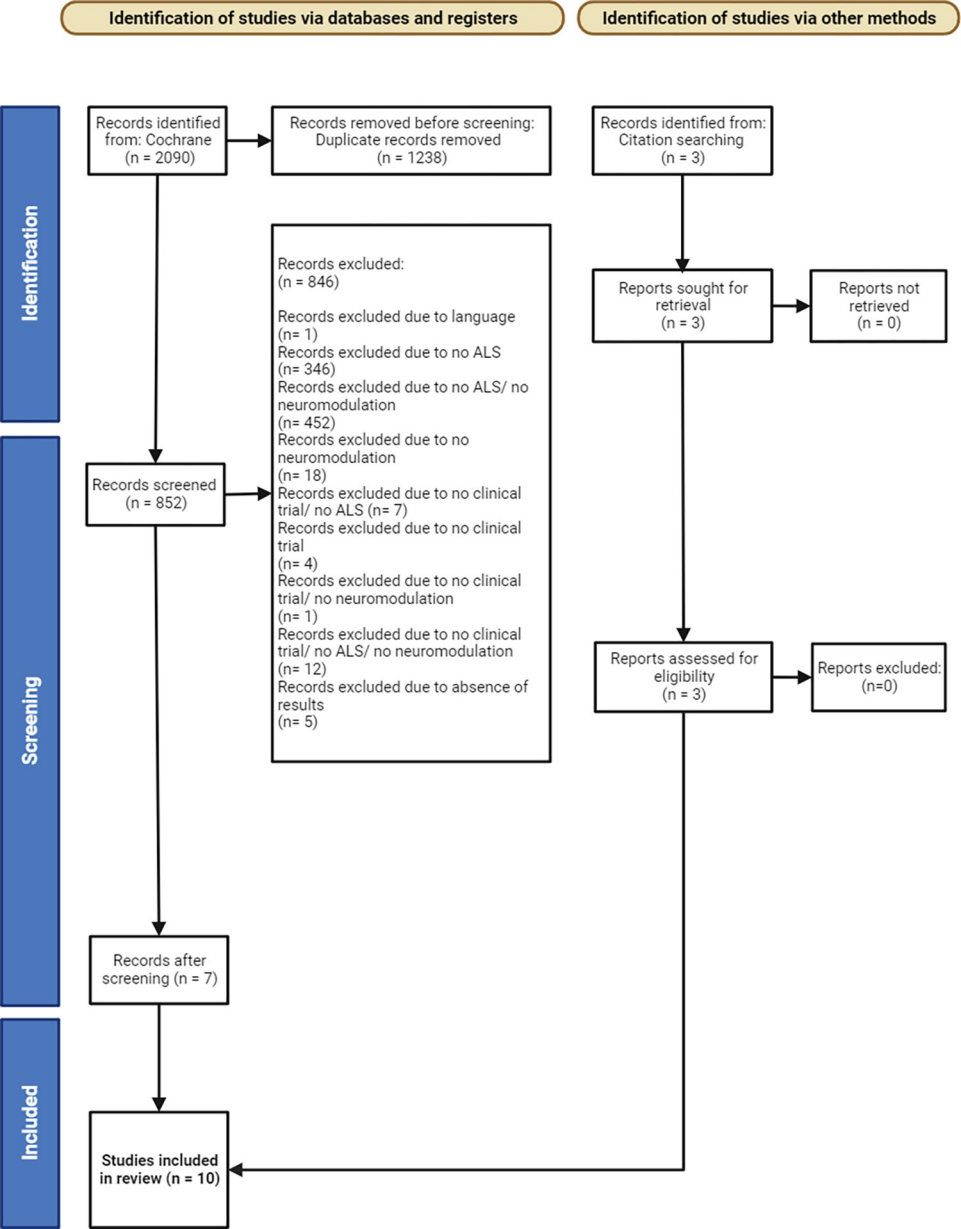
PRISMA workflow diagram for the included references. The inclusion criteria and references added while performing the systematic review have been represented.

This left 6 studies included after screening, with one sought for retrieval. Three other studies were later identified through citation searching and included. This process of study selection ended with a total of 10 studies included as biographical references (Fig 1).

### General characteristics of selected studies

The main characteristics of the included studies are summarised in Table 2. The majority of the studies included are clinical trials with a total of eight (80%), with the exception of two case report (20%) [44, 48]. Different techniques of neuromodulation can be observed within the selected reports. There is a large amount of reports where repetitive transcranial magnetic stimulation (rTMS)(n = 4) was used or continuous theta burst stimulation (cTBS) with transcranial magnetic stimulation (TMS) (n = 1), but there’s also neuromuscular magnetic stimulation (NMMS)(n = 1). There’s also another group of reports that used electrical neuromodulation, with techniques such as cervical transcutaneous spinal stimulation (cTSS)(n = 1), therapeutic electrical stimulation (TES)(n = 1) or transcranial direct current stimulation (tDCS)(n = 1). Finally, low-level laser therapy was also used as a neuromodulation technique (LLLT)(n = 1).

**Table 2:** Publications retrieved concerning neuromodulation technique applied in ALS and their relevant outcomes.

Study	Methodology	Main Findings
Benussi et al. [42]	tDCS	• The muscle strength measured through the MRC scores by the clinician showed significant improvement in the real stimulation group compared to baseline and to sham stimulation. • Significant results were found in the EQ-VAS and the caregiver through the CBI. • Significant differences were observed in SIC and ICF. • Significant correlations were observed between the percentage of improvement in global MRC and CBI scores and the restoration of SICI and between improvement in global MRC and ALSFRS-R scores and the restoration of ICF. • Significant improvement/stabilization in clinical scores of muscle strength, in self-reported quality of life scores, and in proxy-reported caregiver burden, after a two-weeks’ treatment with cortico-spinal tDCS in patients with ALS. And, there were detectable up to 6 months.
Di Lazzaro et al. [32]	rTMS given as cTBS	• cTBS of the motor cortex was performed for five consecutive days every month for one year. Primary outcome was the rate of decline as evaluated with the revised ALSFRS-R. While treatment was well tolerated, there was no significant difference in the ALSFRS-R score deterioration between patients treated with real or placebo stimulation. • The BDNF values are similarly unaffected by a single cycle of rTMS though a slight and non-significant increase was found in the group treated with real rTMS on the third day of stimulation.
Di Lazzaro et al. [22]	rTMS given as cTBS	• Repetitive stimulation of the motor cortex was performed for five consecutive days every month for six consecutive months. Primary outcome measured by ALSFRS-R and MMT. • Both active and sham rTMS patients deteriorated during treatment, however, active rTMS patients showed a modest but significant slowing of the deterioration rate. • No significant difference in BDNF plasma levels between active and sham rTMS patients
Di Lazzaro et al. [54]	rTMS	• No effects of rTMS was observed in transgenic rats overexpressing the human G93A mutant superoxide dismutase 1 gene. • Although the rTMS treatment was well tolerated by the ALS patients, no changes in deterioration were observed. • Patients exposed to low-frequency rTMS showed slower rate of progression during treatment than that evaluated before treatment. • Opposite results were observed in patients exposed to high frequencies.
Handa et al. [48]	TES	• Efficacy in enhancing extremity motion and fostering long-term strength gains. • The application of TES resulted in improved muscle density, whereas the untreated side exhibited signs of deterioration.
Longo et al. [44]	LLLT	• Three cycles of 20 daily sessions at 40 days’ interval (two different wavelengths 810 and 890 nm) showed improved mobility of hands and improved respiratory function after the first cycle. • After the 2nd cycle the patient showed improved strength on upper and lower limbs and improved respiratory function. However, 1 month after the second cycle the general situation showed signs of regression. • During the 3rd cycle increasing improvement was noted for 20 days. It remained so for the following 20 days after which signs of regression started.
Munneke et al. [40]	rTMS given as cTBS	• The amplitude of a single pulse motor evoked potential was significantly decreased (34%) over the days, returning to baseline a week after the last session • The resting motor threshold increased significantly, whereas intracortical inhibition and facilitation did not change over the sessions.
Musarò et al. [45]	NMMS	• Significant effect of rNMMS on MRC scale at the flexor carpi radialis muscle were found, thus demonstrating that the rNMMS significantly improves muscle strength in flexor muscles in the forearm. • The improvement observed in rNMMS-treated muscles was associated to counteracting muscle atrophy, down-modulating the proteolysis, and increasing the efficacy of nicotinic AChRs. • No significant difference was observed in pre- and post-stimulatio CMAP amplitudes, evoked by median nerve stimulation. • Significant down-regulation of MuRF-1 and a reduced trend in atrogin-1 expression in the rNMMS samples post-treatment suggesting that rNMMS preserved muscle mass by modulating protein catabolism. • Significant down-modulation in SREBP-1 in the treated muscle biopsies compared with untreated biopsies confirming that rNMMS counteracts muscle atrophy by down-modulating proteolysis and attenuating the expression of protein synthesis inhibitors. • Significant accumulation of both MiR-24 and MiR-1 in treated muscle compared with untreated muscle. • rNMMS modulated the regulatory circuit of muscle-nerve interplay, up-regulating MiR-206 and down-regulating HDAC4, myogenin as well as the γ and α subunits of the AChR. • Significant down-modulation in Mef2c transcript levels in the rNMMS arm compared with the sNMMS arm.
Wu et al. [47]	cTSS	• More than 170 cTSS sessions were conducted without major safety or tolerability issues. • A cathode-posterior, 2 ms biphasic waveform provided optimal stimulation characteristics. • Responses in bilateral upper extremity muscle responses in subjects with spinal cord injury and ALS. • Resting motor threshold at the abductor pollicis brevis muscle ranged from 5.5 to 51.0 mA. • As stimulus intensity increased, response latencies to all muscles decreased. Homosynaptic post-activation depression was incomplete at lower stimulus intensities, and decreased at higher stimulus intensities.
Zanette et al. [53]	rTMS	• Significant difference at the end of a two-week period of daily 5-Hz rTMS treatment was found for QoL measured through SF-36, maximum voluntary isometric contraction and isokinetic average power when comparing active vs sham treatment. These changes were transitory and outcome measures were not significant two weeks after discontinuation of rTMS.

Note. AChR. Acetilcholine receptors; ALS = Amyotrophic Lateral Sclerosis; ALSFRS-R = ALS functional rating scale; BDNF = Brain-derived neurotrophic factor; CBI = Cambridge Behavioral Inventory; CMAP = compound muscle action potential; cTBS = Continuous theta burst stimulation; cTSS = Cervical transcutaneous spinal stimulation; EQ-VAS = patient self-rated health scale; HDAC4 = Histone Deacetylase 4; LLLT = Low level laser therapy; Mef2c = myocyte enhancer factor 2C; MiR = microRNA; MMT = Manual Muscle Testing; MRC = Medical Research Council; NMMS = Neuromuscular Magnetic Stimulation; rNMMS = Real Neuromuscular Magnetic Stimulation; rTMS = Repetitive Transcranial Magnetic Stimulation; rTMS = Repetitive Transcranial Magnetic Stimulation; sNMMS = Sham Neuromuscular Magnetic Stimulation; SREBP-1 = Sterol regulatory element-binding protein 1; tDCS = Transcranial Direct Current Stimulation; TES = Therapeutic Electrical Stimulation

For the dose and frequency of administration, it varies greatly depending on which neuromodulation technique was used. For TES [48], it was administrated progressively (5 minutes 3 times a day, 7 minutes 6 times a day, 10 minutes 6 times a day and 10 minutes 7 times a day) over the course of 12 weeks. For Cervical transcutaneous spinal stimulation (cTSS) [47], it was administered using constant-current peripheral nerve stimulators (Digitimer DS7A or DS8R) at a regular time of day over the course of more than 170 sessions, with pairs of pulses going from 80% to 200% of RMT delivered at 0.2 Hz in a pseudorandom order 10 times each session.

Regarding Transcranial Direct Current Stimulation (tDCS) [42], 10 sessions of anodal bilateral motor cortex and cathodal spinal tDCS were performed 5 days a week for two weeks followed by another two weeks of 10 sessions of anodal cerebellar and cathodal spinal tDCS 5 days a week with real tDCS for the experimental group and sham tDCS for the sham group, which followed the same pattern and frequency of administration. One study showed the combined application of continuous theta burst stimulation (cTBS) with TMS [40], where participants received 5 sessions of cTBS over five consecutive days.

Furthermore, variations were found in the application of Repetitive Transcranial Magnetic Stimulation (rTMS). Zanette et al. [53] administered a daily dose of 5 Hz sham or active rTMS on a two-week period. However, Di Lazzaro et al. [22] proceeded to do a motor cortex stimulation once a day for five consecutive days every month for a year-long period. Also, Di Lazzaro et al. [54] proceeded to deliver high frequency rTMS to two of their participants (for 2 and 3 months with 2 and 3 cycles of rTMS) and low frequency rTMS to the other two (for 25 and 30 months with 6 and 7 cycles of rTMS). Finally, Di Lazzaro et al. [32] administered daily active and sham rTMS to their participants for five consecutive days monthly for a period of six months.

In the application of Neuromuscular Magnetic Stimulation (NMMS), Musarò et al. [45] proceeded to apply daily sessions of real and sham NMMS (rNMMS and sNMMS) for a period of two weeks with the help of percutaneous needle-biopsy (for 15 of the participants, whilst 7 refused the procedure because of poor compliance) followed by an electrophysiological study of the nicotinic AChRs and a molecular, histomorphometric and histological analysis of the muscle samples.

Regarding photobiomodulation (PBM), Longo et al. [44] applied three cycles during ten consecutive days, separated by a forty day interval each, with two daily sessions where two different lasers, a 810 diode laser (wavelength of 810nm, average power density of 30 W/cm2 in continuous mode, maximum fluence oscillating between 12 and 15 J/cm2 and spot size of 5 cm) and a 890 diode laser (wavelength of 890nm, peak power of 10 W/cm2 in pulsed mode, pulse frequency of 250 Hz, maximum fluence of 4 J/cm2 and spot size of 1 cm) coupled with a magnetic field (14Hz, 10 mT) were applied.

Regarding the sample sizes, they varied between 1 to 30 participants, with two case reports that only have one [44, 48] (20%), two clinical trials with 4 participants [46, 54] (20%) and the rest with between 10 [55] and 30 participants [42] (60%). Also, the general outcomes varied depending on what neuromodulation technique was used and the length of the protocol. When TES was applied [48] a melioration of the motion in the extremities and an increase of strength after a long term application of the treatment was observed. The muscle thickness showed improvement as well in comparison with the untreated side which showed a deterioration in muscle atrophy. Data from the application of cTSS [47] showed no significant improvement in motor threshold and muscle activation, activating mainly afferent sensory circuits that are not affected by ALS. tDCS [42] showed significant improvement in muscle strength, quality of life and proxy-reported caregiver burden after two weeks of treatment.

Moreover, the combination of cTBS with TMS, Munneke et al. [40] showed a depressing effect on corticospinal excitability by 34% over 5 sessions and a significant increase in resting motor threshold, whereas no significant changes in intracortical inhibition and facilitation were found.

Musarò et al. [45] showed a significant increase in muscle strength and a significant decrease in muscle atrophy which suggests an improvement in fine motor skill when applying NMMS. Similar results of improvement were found with PBM [44] which resulted in improvements in mobility of hands, respiratory function, strength in upper and lower limbs during the first two cycles. However, this improvement was followed by a regression a month following the end of said cycle, with another twenty days’ period of improvement during the third and last cycle that was followed by signs of regression within twenty days later.

Finally, rTMS application Zanette et al. [53] showed that 5 Hz rTMS improves motor function in the experimental group, but specified that these outcomes need confirmation due to the fact that these are preliminary results. The trial of Di Lazzaro et al. [22] showed that use of cTBS with rTMS can be beneficial in the early stages of the onset symptoms, and not when it progresses which was shown with participants´ scores declining after a year of treatment. The trial of Di Lazzaro et al. [54] showed a slowed progression of onset symptoms in participants exposed to low-frequency rTMS. Also, the clinical trial of Di Lazzaro et al. [32] showed a significantly slower rate of decline of both functionality and manual muscle testing after stimulation of the motor cortex with cTBS and rTMS.

### Meta-analyses

Of the included papers, meta-analysis was conducted on muscle strength, which was evaluated with Manual Muscle Testing (MMT), on maximum voluntary isometric contraction (MVIC), and on ALS functionality, which was assessed by the ALS functional rating scale (ALSFRS-R). Also, brain-derived neurotrophic factor (BDNF), short-latency intracortical inhibition (SICI), intracortical facilitation (ICF), resting motor threshold (RMT), and quality of life (QoL) were analysed (Table 2).

**Fig 2:**
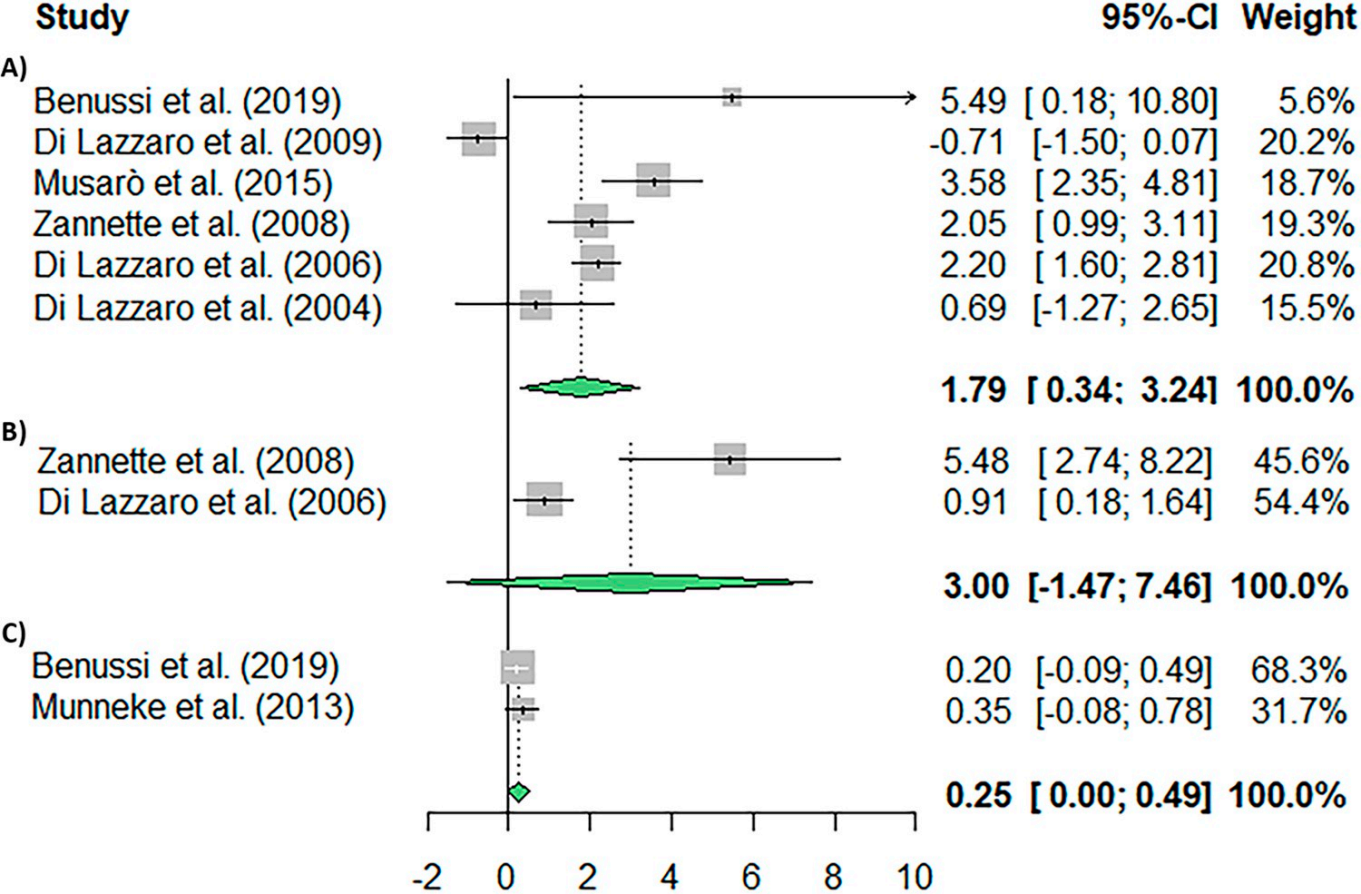
Meta-analysis using a random effects model of selected studies relating to MMT, MVIC and RMT. (**A**) Shows the meta-analysis for muscle strength measured by manual muscle testing (MMT) (p = 0.012) (**B**) Shows the meta-analysis for maximum voluntary isometric contraction (MVIC) (p = 0.1883). (**C**) Shows the meta-analysis for resting motor threshold (RMT) (p = 0.0457). The plot shows the effect estimates and corresponding confidence intervals (CI) for each study included in the meta-analysis. The relative weight or contribution of each study to the overall effect estimate is also included in percentages. The overall weighted effect is indicated by a diamond at the bottom of the figure. The figure was generated with *R* software version 4.3.1.

A meta-analysis was conducted using the random effects model to assess the relationship between muscle strength and ALS in patients diagnosed with the disease. Muscle strength, understood as the ability of muscles to contract and overcome resistance, was measured with the Manual Muscle Testing (MMT) in all studies included in the meta-analysis (k = 6) (Fig 2A). We found that the studies were highly heterogeneous (I^2^ = 83.18%; p = 0.001). However, Kendall’s Tau (0.200; p = 0.719) and Egger’s Regression (1.429; p = 0.153) suggested that publication bias may not be a major concern in this analysis. Nevertheless, significant differences were seen in muscle strength (p = 0.012).

Regarding the maximum voluntary isometric contraction (MVIC), it is a standardised method for the measurement of muscle strength in patients with neuromuscular disease. Two studies were included in this meta-analysis (k = 2). Our results showed heterogeneity in the assessed studies (I^2^ = 89.96%; p = 0.0016), and no significant differences were found in this variable (p = 0.1883). Lastly, the resting motor threshold (RMT), defined as the minimal stimulus intensity that generated a minimal motor-evoked response (approximately 50 μV in a minimum of 5 out of 10 trials) while at rest, was assessed in two of the included studies (k = 2). The results showed no significant differences in the heterogeneity between the studies (I^2^ = 0.00%; p = 0.5732), although significant differences were found in the RMT (p = 0.0457).

**Fig 3:**
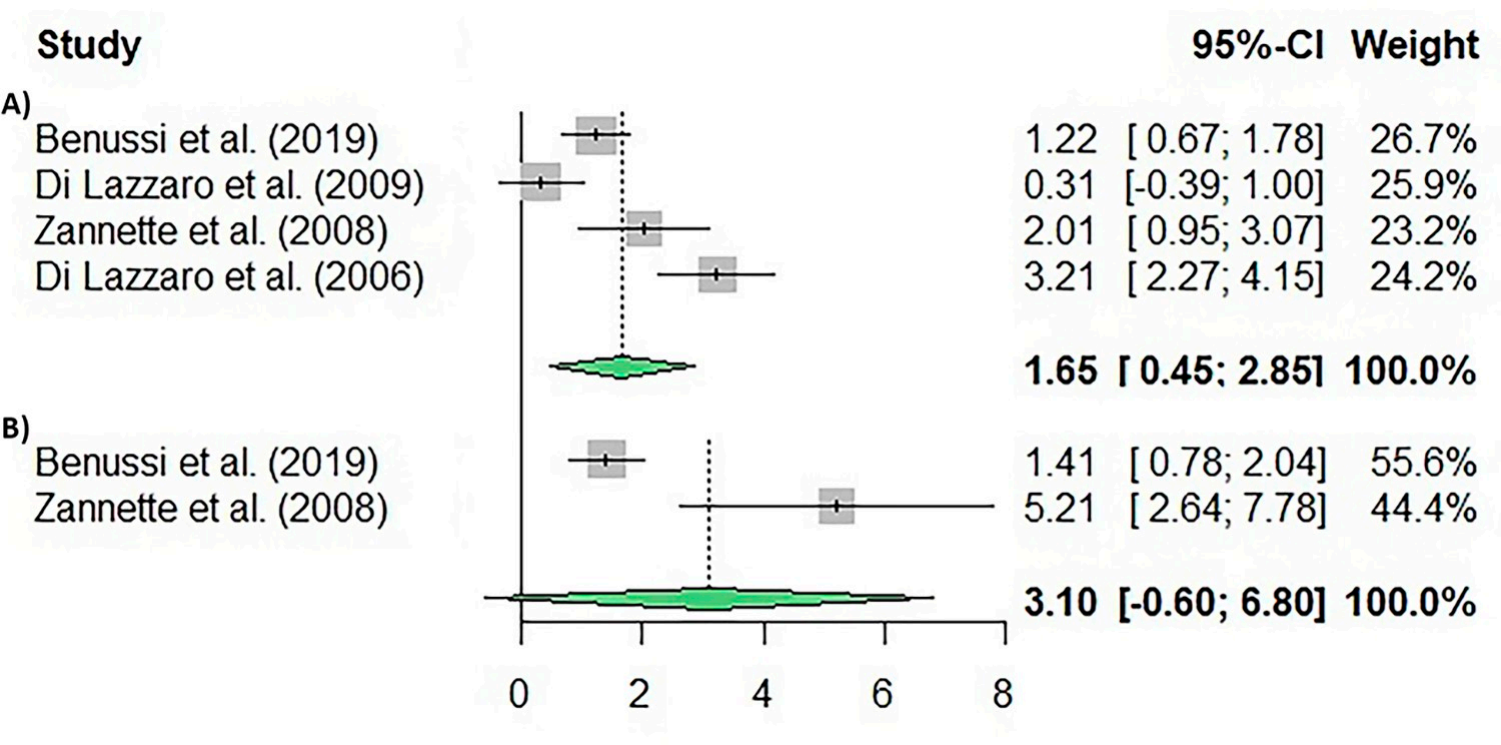
Meta-analysis using a random effects model of selected studies relating to ALSFRS-R (**A**) and quality of life (B). The plot shows the effect estimates and corresponding confidence intervals (CI) for each study included in the meta-analysis. The relative weight or contribution of each study to the overall effect estimate is also included in percentages. The overall weighted effect is indicated by a diamond at the bottom of the figure. The figure was generated with *R* software version 4.3.1.

When assessing functionality in ALS through the ALSFRS-R, which allows to measure disease progression in patients with ALS (k = 4) (Fig 3A), no significant heterogeneity was found (I^2^ = 73.21%; p = 0.0013) among the studies. Moreover, significant differences were found in the analysed variable (p = 0.007).

Quality of life (QoL) can be described as the overall sense of well-being experienced by either a population or an individual. It encompasses both positive and negative aspects of their existence at a particular moment. In the assessed studies, the assessment of QoL was conducted using the SF-36 and 5Q-5D-5L scales (k = 2). Our results showed heterogeneity in the assessed studies (I^2^ = 95.85%; p = 0.0001), and no significant differences were found in this variable (p = 0.5909).

**Fig 4:**
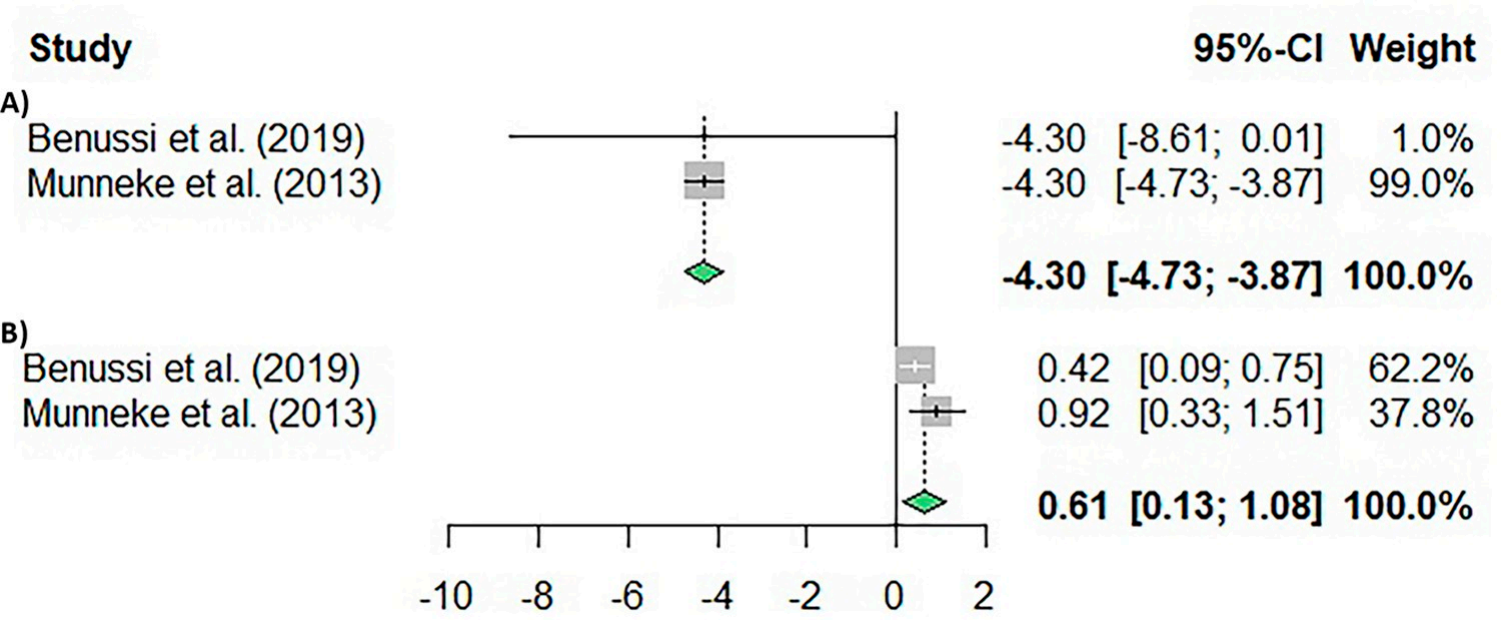
Meta-analysis using a random effects model of selected studies for the SICI (p = 0.0001) (A) and ICF (p = 0.1338) (B) variables assessed in the studies included. The plot shows the effect estimates and corresponding confidence intervals (CI) for each study included in the meta-analysis. The relative weight or contribution of each study to the overall effect estimate is also included in percentages. The overall weighted effect is indicated by a diamond at the bottom of the figure. The figure was generated with *R* software version 4.3.1.

Short-interval intracortical inhibition (SICI), which is an inhibitory phenomenon occurring in the motor cortex (M1) used in studies that use TMS (k = 2; Fig 4A), was also assessed. Our results showed no significant differences in the heterogeneity of the assessed studies (I^2^ = 0.00%; p = 1.000), whereas significant differences were found in SICI (p = 0.0001).

Also, intracortical facilitation (ICF), which is an excitatory phenomenon occurring in the motor cortex (M1) used in studies that use TMS, was assessed (k = 2; Fig 4B). The results showed no significant results for heterogeneity between the included studies (I^2^ = 52.44%; p = 0.1470), and no significant differences were found in ICF (p = 0.1338).

**Fig 5:**
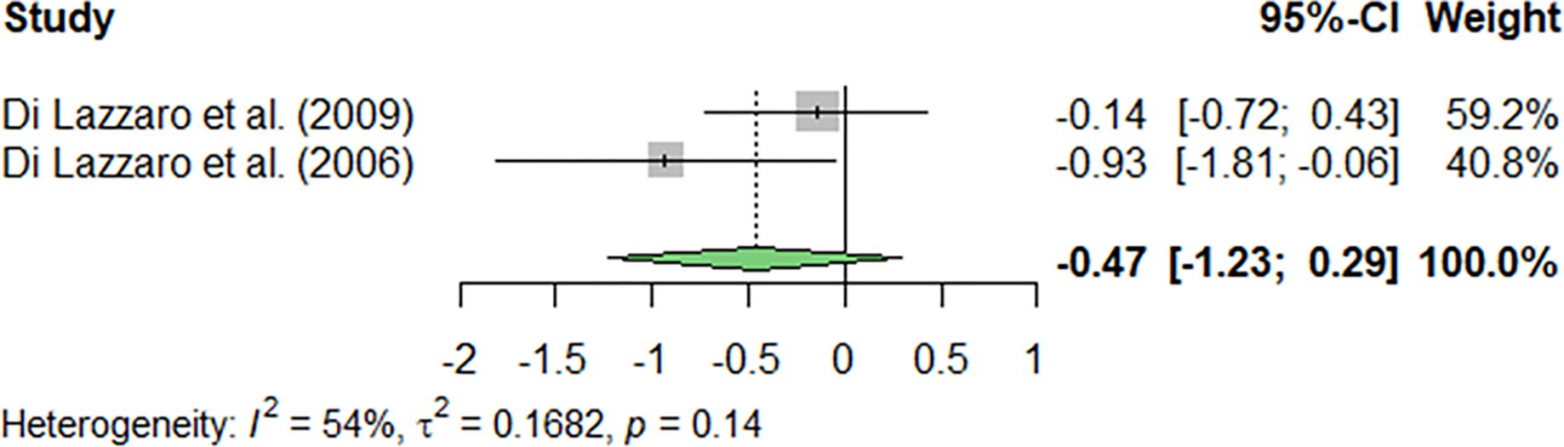
Meta-analysis using a random effects model of selected studies relating to BDNF variables assessed in the studies included. The plot shows the effect estimates and corresponding confidence intervals (CI) for each study included in the meta-analysis. The relative weight or contribution of each study to the overall effect estimate is also included in percentages. The overall weighted effect is indicated by a diamond at the bottom of the figure. The figure was generated with *R* software version 4.3.1.

Finally, our exploration extended to the influence of brain-derived neurotrophic factor (BDNF), a biomarker associated with neurodegeneration that contributes to the diagnosis of ALS. This factor was taken into account in two studies that were incorporated into the meta-analysis (k = 2). Our results showed no significant differences in the heterogeneity of the studies (I^2^ = 53.94%; p = 0.1406), and no significant differences were found in BDNF (p = 0.2297; Fig 5).

### Method quality assessment

The quality of the included studies was generally medium. Three of the eight clinical research studies included in this review were affected. Two of them were due to the selected groups being non-equivalent and thus preventing random assignment to the different groups. For example, Wu et al. [47] selected patients with different pathologies (ALS, brain damage, or healthy controls), while Munneke et al. [40] selected healthy patients without ALS as controls. Similarly, five of eight records didn’t mention if participants or personnel were blinded during the experiment. In these records, the authors did not mention if there was a blinding of outcome assessment. Regarding the justification of missing data, six of the 10 included references justified missing data. Also, six of the 10 references included non-significant data, with the study by Di Lazzaro et al. [22] being the only one that did not present data for those variables that were not significant. All studies mentioned the presence of possible limitations that could affect the methodological quality of the study (see Table 3 for clinical research studies).

**Table 3:** Included clinical research studies rated against the Cochrane Risk of Bias tool.

Authors	Random sequence generation	Allocation concealment	Blinding of participants and personnel	Blinding of outcome assessment	Incomplete outcome data	Selective reporting	Other bias
Benussi et al. [42]	+	?	+	+	+	+	+
Di Lazzaro et al. [32]	+	+	+	+	+	-	+
Di Lazzaro et al. [22]	+	+	+	+	?	+	+
Di Lazzaro et al. [54]	?	?	?	?	?	+	+
Munneke et al. [40]	-	?	?	?	+	+	+
Musarò et al. [45]	+	+	+	+	+	+	+
Wu et al. [47]	-	-	-	?	+	?	+
Zanette et al. [53]	+	?	?	?	?	+	+

Note. + = High risk of bias;— = Low risk of bias;? = Unclear risk’ of bias.

Finally, the case reports of single cases were evaluated with the CaSE. Handa et al. [48] scored 9/18, with the bias being especially affected due to the lack of justifications in the case report or a factor that could facilitate the comprehension of the case. Similarly, the authors did not mention the information according to the data analysis, and the limitations are not discussed in the paper. Longo et al. [44] showed a score of 11/18, with the bias especially affecting the justification of the case, the recompilation of data, and the lack of data analysis.

## Discussion

Amyotrophic lateral sclerosis (ALS) is a neurodegenerative condition typified by the gradual degeneration of both upper motor neurons (UMN) and lower motor neurons (LMN) within the motor cortex, brainstem, and spinal cord. Nowadays, ALS is recognized to extend beyond being solely a motor neuron disorder, as evidenced by the fact that 15–50% of patients experience cognitive disturbances. These disturbances vary, ranging from cognitive impairment to the more characteristic frontotemporal lobar dementia (FTLD), which is associated with damage to the frontal and temporal cortices [55]. The prognosis for ALS is uniformly fatal, with a median survival duration from the onset of initial symptoms to death typically lasting around 36 months [56].

Currently, dependable diagnostic and prognostic biomarkers for ALS are lacking. The diagnostic process hinges on the elimination of conditions that mimic ALS [57]. As of now, diagnostic certainty continues to rest largely on neuropathological hallmarks, contingent upon the identification of inclusion bodies within the cytoplasm of lower motor neurons in the spinal cord and brainstem [58]. In this scenario, only two drugs are currently labelled for ALS: riluzole and edaravone which is available in only a few countries worldwide (USA, Canada, Japan, and Switzerland in Europe) [15, 59]. Given that the therapeutic impact of these drugs remains relatively modest, resulting in only a marginal extension of survival by a few months, there exists an urgent requirement for novel treatments that possess the potential to significantly alter the natural progression of the disease.

Neuromodulation, on the other hand, has emerged as a potentially more specific form of treatment without the systemic side effects of pharmacotherapies. Neuromodulation interventions use electrical, magnetic and photonic stimulation to modulate neuronal activity and elicit a therapeutic response. These procedures are generally adjustable, reversible, and have demonstrated some efficacy in the treatment of several conditions; however, its short and long-term effects when applied to ALS are scarce and a broad overview of their impact has been addressed in this study.

### Effects of electrical stimulation on ALS

The application of Transcranial Electrical Stimulation (TES) utilizing intramuscular electrodes has revealed two distinct therapeutic effects on paralyzed extremities affected by upper motor neuron disorders. These effects encompass both efferent and afferent outcomes. Efferent effects, brought about by the stimulation of alpha-motoneurons, manifest as increases in muscle volume and force for the stimulated muscles [60, 61]. Indeed, our meta-analysis provides evidence linking muscle strength to ALS progression after TES (Fig 2). Conversely, afferent effects are achieved through the stimulation of afferent nerves originating from the muscle, fascia, and/or tendon. Since the threshold of g1a afferent fibers for electrical stimulation is lower than that of alpha-motoneurons, a stimulus current that triggers muscle contraction invariably also prompts afferent volleys in g1a afferent fibers. Of note, the afferent effects tend to manifest more rapidly compared to the efferent effects. This dynamic results in the reduction of spasticity, primarily through the mechanism of reciprocal inhibition wherein the antagonist of the activated muscle is inhibited.

Based on this principle, Handa et al. [48] applied TES to ALS patients demonstrating effectiveness in augmenting extremity motion and promoting sustainable strength improvements. Also, the utilization of TES led to enhanced muscle density. In this line, Benussi et al. [42] aimed to elucidate the lasting effects of repeated sessions of transcranial direct current stimulation (tDCS) that involved concurrent bimodal stimulation of the motor cortex and cathodal stimulation of the spinal cord (referred to as cortico-spinal tDCS) in individuals diagnosed with ALS, both in terms of their clinical condition and the intracortical neural responses. To achieve this, a randomized, double-blind, and sham-controlled clinical trial was conducted. Each participant underwent a structured evaluation at various time points such as baseline (pre-stimulation), after two weeks of either real or sham tDCS (post-stimulation), two months’ post-intervention, and six months into the follow-up period. Significant results were observed in clinical outcomes such as the patient self-rated health scale (using EQ-VAS), and the caregiver (via CBI), whereas no changes were observer by the clinician assessment (using MRC score). These observed changes, which remained discernible for a period of up to 6 months, were found to be associated and interrelated with the restoration of measures concerning intracortical circuits. Specifically, measures of short interval intracortical inhibition (SICI) and intracortical facilitation (ICF) exhibited correlations. SICI, regarded as indicative of short-lasting postsynaptic inhibition mediated through GABA-A receptors at the local interneuron level, has been previously demonstrated to be altered in ALS. Moreover, it has been shown to correlate with disease progression and survival [62]. Conversely, ICF assumed to signify net facilitation, likely mediated by glutamatergic NMDA receptors, has been indicated to be increased in ALS [63, 64]. This potentially positions ICF as an indirect marker of excessive glutamate activity within the context of ALS. Furthermore, the conducted meta-analysis aimed at evaluating the measure’s relevance to the observed alterations in ALS following transcranial Direct Current Stimulation (tDCS) demonstrated the statistical significance of SICI whereas no evidence was found for the ICF.

Moreover, variations in the placement of the electrical stimulation have been tried in ALS motivated by several studies where the delivery of electrical stimulation over the epidural surface of the lumbar spinal cord has produced improvements in motor and cardiovascular function [65–70]. So, Wu et al. [47] tried a novel electrode configuration for the administration of non-invasive phasic cervical transcutaneous spinal stimulation (cTSS) which involves placing the anode over the midline of the anterior surface of the neck, positioned several segments rostral to the cathode, which is placed posteriorly. As suggested by the authors, the focused stimulation of the spinal cord has the potential to engage inherent neural circuitry that facilitates the restoration of natural movement synergies. This process can trigger the re-expression of movement patterns that emulate those occurring naturally [71, 72]. Indeed, their results showed that cTSS possesses the capability to initiate motor responses through the activation of afferent sensory circuits that remain unaffected in individuals with ALS. This phenomenon aligns with the hyperreflexic responses often observed upon clinical examination, such as the hyperactive responses to tendon stretch. Also, the cathode-posterior, anode-anterior configuration utilized in cTSS appears to have the capacity to stimulate both dorsal afferent and ventral efferent root fibers. This observation was supported by two key observations. Firstly, there is an intensity-dependent alteration in the latency of muscular responses. Secondly, there is a partially intensity-dependent modification in post-activation depression (PAD), PAD decreased at higher stimulus intensities. The occurrence of homosynaptic PAD, wherein presynaptic large-diameter afferents struggle to release sufficient neurotransmitter in rapid succession, further supports that cTSS effectively triggers efferent nerve roots situated 2 to 4 cm distal to motor neuron cell bodies, independently of synaptic connections. Hence, elevated levels of PAD suggest the activation of motor neurons via large-diameter afferents in a transsynaptic manner. Conversely, lower levels of PAD suggest non-synaptic activation of efferent motor axons [73–75].

### The long-term effects of TMS

It was the pioneer study by Di Lazzaro et al. [32] which showed that there were no discernible effects of repetitive Transcranial Magnetic Stimulation (rTMS) in transgenic rats that overexpressed the human G93A mutant superoxide dismutase 1 gene. Also, when applied to ALS patients, the ones subjected to low-frequency rTMS exhibited a slower rate of disease progression during the treatment phase compared to the assessment conducted prior to treatment initiation. Conversely, patients exposed to high-frequency rTMS displayed opposing outcomes. The authors proposed that low-frequency rTMS induces a reduction in motor cortex excitability which could potentially mitigate the excessive activation of glutamate receptors, thus attenuating the glutamatergic excitotoxicity observed in ALS patients. By this mechanism, high-frequency rTMS might potentially enhance the activation of glutamatergic receptors within the motor cortex, thereby augmenting glutamatergic excitotoxicity.

Owing to the limited size of the ALS patient cohort in the aforementioned investigation, the authors aimed to replicate the study utilizing a more substantial sample size. Furthermore, they sought to extend the follow-up period to encompass six months [22] and one year [32]. Both studies administered a novel paradigm of rTMS termed continuous theta burst stimulation (cTBS) [76, 77] of the motor cortex for five consecutive days every month for the mentioned periods. The results in the six-months follow-up study showed that although active and sham rTMS patients deteriorated during treatment, active rTMS patients showed a modest but significant slowing of the deterioration rate. However, the one-year follow up study did not show any significant differences in the revised ALS functional rating scale (ALSFRS-R) between real or placebo stimulation. Interestingly, a meta-analysis conducted to assess the relevance of ALSFRS-R (Fig 3) as a variable to track the impact of rTMS on ALS progression found significant differences. These results contradicted the positive findings from Di Lazzaro et al. [32] using the same technique of stimulation, that showed a modest but significant slowing of the deterioration rate in ALS patients treated with real cTBS for six months. One of the main differences found between the studies is the disease severity at baseline, with patients included in these two studies presenting a more advanced progression of the disease. Also, it is important to note that alongside the long-lasting decline in the excitability of excitatory cortical circuits, cTBS also induces a reduction in the excitability of intracortical inhibitory circuits. This reduction is illustrated by a decrease in short latency intracortical inhibition, which is commonly considered an indicator of GABA-A activity [77]. Given that functional alterations in the activity of intracortical inhibitory circuits have been documented in ALS patients [78], it is plausible that some of the observed effects of real cTBS in these patients with advanced disease progression might be attributed to the modulation of these intracortical inhibitory circuits.

In order to disentangled the effects of cTBS on the modulation of these circuits, Munneke et al. [40] explored corticospinal excitability in individuals with ALS following cTBS. Both the immediate impact of a single session and the long-term effects following repeated administration were investigated. Their results showed that whereas a single session of cTBS has inhibitory properties on corticospinal excitability in healthy subjects, those results were not found in ALS patients unless the stimulation was repeated over 5 days. The authors provided rationale for these outcomes based on two mechanisms. Firstly, they proposed the possibility of an accumulating effect of cTBS supported by new gene expression and synaptic alterations [56]. Secondly, they postulated an influence of synaptic plasticity, related to the concept of metaplasticity which has been linked to changes in NMDA receptors [79, 80]; however, cTBS seems to activate non-NMDA glutamatergic connections of the motor cortex [81, 82], giving more support to the first postulated mechanism.

Moreover, the abovementioned studies evaluated the effects of cTBS on BDNF production, which is a potent survival factor for motoneurons, as a possible marker of cTBS effects on ALS improvement [22, 42]. Both studies were accompanied by the absence of differences in BDNF plasma levels between active and sham rTMS ALS patients after a single cycle of five days of cTBS. Also, the meta-analysis revealed no significant differences in this variable. Two plausible explanations could be provided, firstly that BDNF plasma levels might not be correlated to the BDNF level in the brain; secondly, that BDNF changes over time could shed more light into the response of ALS to cTBS.

Whereas those studies focused on cognitive and molecular changes, Zanette et al. [53] explored the impact of a two-week regimen of daily 5-Hz rTMS, in patients with ALS, on Quality of Life (QoL), as assessed by SF-36, maximum voluntary isometric contraction, and isokinetic average power. The authors observed some transient improvements with outcome measures losing their significance two weeks after the cessation of rTMS. This is in line with the results found in the meta-analysis where not significant differences were found in QoL and voluntary isometric contractions (Fig 2). So, those findings could not only be supported by the short-term production of new proteins but also by the emotional enrolment of the ALS patients on a clinical trial.

Finally, Musarò et al. [45] approached the treatment of ALS at the neuromuscular level through the NMMS which does not activate nociceptors [83]. Their results showed changes in the modulation of muscle protein catabolism evidenced by a down-modulation of MuRF-1 and atrogin-1 expression, as well as a decrease in the expression of protein synthesis inhibitors such as SREBP-1. Moreover, an augmentation of factors associated with the homeostatic preservation of skeletal muscle, specifically MicroRNAs (MiR-24 and MiR-1), was identified. Due to the effect of repetitive NMMS on mitigating muscle atrophy in ALS patients, the electrophysiological function of nicotinic acetylcholine receptors (AChRs), which play a pivotal role in muscular contraction, was also studied. Their results showed that rNMMS up-regulates MiR-206, which in turn modulates HDAC4, myogenin and AChRγ, all of which act as important regulators of the signaling that detects nerve activity within the muscle. So, local administration of rNMMS prevents muscle atrophy, maintains fiber type composition, and stabilizes the neuromuscular junction. Consequently, it contributes to enhancing the overall resilience and strength of muscles in patients with ALS.

### Photobiomodulation as a novel approach to ALS

Excitotoxicity further worsens the mitochondrial dysfunction in neurodegenerative diseases, causing an intramitochondrial Ca2+ overload and triggers apoptosis by the release of CCO through the mitochondrial transition pore. Mitochondrial dysfunction hence appears to be a central mediator of neurodegenerative disease pathogenesis and disease progression [84–86] such as ALS. Under this scenario of the potential need to improve mitochondrial function to delay neurodegenerative progression, photobiomodulation (PBM) appears as a non-pharmacological therapeutic approach that involves using red or near-infrared wavelengths (650–1200 nm) which are absorbed by chromophores present in cells. Specifically, the CCO, mitochondrial electron chain transport complex IV absorbs wavelengths from 600 to about 900 nm. Moreover, PBM therapy has the remarkable ability to stimulate stressed neurons to produce essential neurotrophic factors like brain-derived neurotrophic factor (BDNF) and glial cell line-derived neurotrophic factor (GDNF) [87–89].

In this line, Longo et al. [44] applied different wavelengths (810nm and pulsed-890 nm coupled with magnetic field) to an ALS study case. Three cycles of 20 daily sessions at 40 days’ interval were given. The authors found that one month after the completion of the second cycle, a decline in the overall condition was observed. However, during the third cycle, notable enhancements were observed over a period of 20 days. This positive trend persisted for the subsequent 20 days, after which indications of regression commenced. However, the interpretations of these results are difficult, not only for the nature of the case reports, but also because of the physiotherapy rehabilitation paired with the treatment and the combination of pulsed and single PBM treatment together with magnetic stimulation. Although promising, further research should be done on the improvements related to PBM application in ALS.

### Limitations and future directions

Similar to most medical interventions, neuromodulation carries inherent risks. Therefore, it is imperative to substantiate that its actual efficacy prevails over potential adverse outcomes. Moreover, the utilization of neuromodulation devices entails relatively substantial financial expenditures, encompassing expenses associated with device acquisition, battery replacement, and potential complications or device malfunctions [90]. For a novel pharmacological treatment to attain approval, it must demonstrate benefits that surpass placebo effects, biases, and symptom fluctuations. However, these stringent prerequisites are not yet universally applied to treatments involving neuromodulation. Before extending the application of neuromodulation therapy, it is pivotal to rigorously evaluate its treatment effects.

Another important point to mention when applying neuromodulation across the studies is the effectiveness of neurostimulation frequencies compared to placebo which has exhibited variability among different studies, thereby casting doubt on the superiority of active neurostimulation over placebo. Discrepancies between these trials may have arisen due to biases introduced by patients becoming unblinded. The prevailing perception within the neuromodulation literature suggests that comparing these procedures to placebo controls may not be feasible or necessary. Nonetheless, the ongoing evolution of neuromodulation techniques, which now facilitate the inclusion of robust placebo controls, challenges this assumption. Furthermore, investigations into so-called placebo-like effects [91] can be undertaken through the differentiation between open and hidden administration of active treatment. In designs characterized by open–hidden administration, patients solely receive active treatment, thereby eliminating the administration of inactive placebo treatment. Similar designs might offer a pathway to incorporate placebo controls within patients undergoing neuromodulation device treatment.

Also, almost all ALS patients involved in the studies were concurrently using riluzole, a medication recognized for its modulation of excitatory neurotransmission or edaravone which mechanism are still unknown. The prolonged use of those medications could impact on cortical excitability altering the effects of cTBS or tDCS on ALS. So, further investigations are warranted to delve into the influence of riluzole or edaravone on the after-effects of these neuromodulatory techniques.

Furthermore, as outlined in a recent publication by Di Lazzaro et al. (2024) [92], various considerations pertaining to experimental design, including statistical power influenced by sample size and study variability, differential utilization of indexes for endpoint selection, and factors associated with the intrinsic pathophysiological changes in the disease, such as distinct stages of disease progression, the presence of extra-motor involvement, and the neurobiological impacts of brain stimulation interventions, must be considered when formulating protocols and techniques for achieving the intended neuromodulatory effects in this population. Finally, Dubbioso et al. [93] performed a longitudinal study aimed to investigate the association between autonomic dysfunction and disease progression and survival in ALS. The study included newly diagnosed ALS patients and a healthy control group, assessing autonomic symptoms through a dedicated questionnaire and parasympathetic cardiovascular activity through heart rate variability (HRV). Results showed that ALS patients experienced more autonomic symptoms, particularly in bulbar onset cases, and these symptoms increased over time. Higher autonomic symptom burden was independently associated with faster development of King’s stage 4, while urinary complaints were linked to shorter survival. HRV in ALS patients was lower than in controls and further decreased over time, indicating a progressive parasympathetic hypofunction. This suggests that autonomic dysfunction is an intrinsic non-motor feature of ALS, and a greater autonomic burden is a negative prognostic factor for disease progression and survival. In this context, vagus nerve stimulation (VNS) which is a neuromodulatory technique that involves the targeted application of electrical impulses to the vagus nerve, a key component of the autonomic nervous system could play a crucial role in regulating various physiological processes, including heart rate, respiratory function, and gastrointestinal activity which are altered in ALS. To date, promising results applying VNS have been observed in Parkinson´s disease (PD). In an open-label pilot study involving 19 patients with PD-related disorders, including twelve with freezing of gait (FoG), the impact of single-dose non-invasive vagus nerve stimulation (nVNS) on gait patterns was investigated. The study applied two treatments to the left vagus nerve in the left side of the neck, and assessments were conducted before and 15 minutes after nVNS application [94]. The results demonstrated improvements in spatiotemporal gait parameters, including step count, velocity, step length, and stride velocity variability. Video analysis of FoG patients revealed enhancements in turning time, steps taken for turning, and steps taken for start hesitation. A subsequent crossover randomized controlled study confirmed these initial findings, showing significant improvements in walking speed, stance time, step length, and overall motor function during the active phase of nVNS stimulation compared to sham stimulation [95]. The average duration of freezing episodes was reduced, although other FoG measures remained unchanged. ALS is characterized by progressive motor neuron degeneration, leading to muscle weakness and impaired motor function. Therefore, the encouraging results in gait parameters and overall motor performance suggest that exploring nVNS in ALS may have potential therapeutic implications, potentially enhancing mobility and mitigating motor symptoms in ALS patients. Further research is warranted to specifically investigate the application of nVNS in the context of ALS and assess its impact on motor function and overall disease progression in this population.

## Conclusion

Amyotrophic lateral sclerosis (ALS) is a devastating neurodegenerative disease that affects both upper and lower motor neurons. Although it used to be considered a motor neuron-centred disease, it has been increasingly recognised that ALS may also be associated with cognitive impairment, ranging from cognitive decline to frontotemporal lobar dementia.

Despite the severity of the disease and its lack of effective treatment options, advances in neuromodulation are offering new perspectives. Electrical and transcranial magnetic stimulation have demonstrated potential therapeutic effects in ALS patients. Electrical stimulation can have both efferent (on alpha motor neurons) and afferent (on afferent nerves) impacts, which may result in improvements in muscle strength and a reduction in spasticity. Low-frequency repetitive magnetic stimulation has also shown mixed results, with some studies suggesting a decrease in disease progression. Photobiomodulation has emerged as a novel approach which results are promising in terms of temporary improvements in quality of life and muscle function.

## Supporting information

S1 Text PRISMA 2009 checklist. (PDF)

S1 Code Detailed code in R to calculate size effect from F-statistic. (DOCX)

S2 Code Detailed code in R to perform the meta-analysis with forest plot. (DOCX)

S3 Code Detailed code in R to calculate the fail-safe N based on the Rosenthal approach. (DOCX)
