# Supplementary material for: Current perspectives on neuromodulation in ALS patients: A systematic review and meta-analysis
Source: PLoS One. 2024 Mar 29;19(3):e0300671. doi: 10.1371/journal.pone.0300671 (PMC10980254; doi:10.1371/journal.pone.0300671)
Supplement: S1 Code — (DOCX) [file pone.0300671.s002.docx]

# Define the values

F_interaction <- XX # F of the interaction

df1 <- X # Degrees of freedom of the numerator of the F

df2 <- X # Degrees of freedom of the denominator of the F

# Calculate the effect size d

d <- sqrt((F_interaction * (df1 + df2)) / (df1 * (df2 - 2)))

# print the result

cat("The effect size d calculated from the F of the interaction is:", d)
