# Supplementary material for: Current perspectives on neuromodulation in ALS patients: A systematic review and meta-analysis
Source: PLoS One. 2024 Mar 29;19(3):e0300671. doi: 10.1371/journal.pone.0300671 (PMC10980254; doi:10.1371/journal.pone.0300671)
Supplement: S2 Code — (DOCX) [file pone.0300671.s003.docx]

# Install and load the metafor package install.packages

("metafor ")

library(metafor)

# Create a data frame with the study data

data <- data.frame(

Study = c(“XX”, "XX"),

Size Effect = c(XX, XX),

Sampling Variances = c(XX, XX)

)

# Perform meta-analysis analysis

meta_result <- rma.uni(Size Effect, Sampling Variances, data = data)

# Print the results of the meta-analysis analysis

print(meta_result)

# Create the forest plot with the names of the studies and weights

forest(meta_result, slab = data$Study, showweights = TRUE)
