# Supplementary material for: Current perspectives on neuromodulation in ALS patients: A systematic review and meta-analysis
Source: PLoS One. 2024 Mar 29;19(3):e0300671. doi: 10.1371/journal.pone.0300671 (PMC10980254; doi:10.1371/journal.pone.0300671)
Supplement: S3 Code — (DOCX) [file pone.0300671.s004.docx]

# Load the metafor library

library(metaphor)

# Create a dataframe with the data

data <- data.frame(

Study = c("Study 1", "Study 2", "Study 3"),

Size Effect = c(XX, XX, XX),

Sampling Variances = c(XX, XX)

)

# Filter the data to remove rows with NA values

data <- data[complete.cases(data), ]

# Calculate the Fail-Safe N based on the Rosenthal approach

fsn_result <- fsn(yi = data$ Size Effect, vi = data$ Sampling Variances)

# Print the result of Fail-Safe N

print(fsn_result)
